# Point-of-Care Ultrasound Assists in Rapid Diagnosis of T-cell Lymphoblastic Lymphoma in a Young Boy

**DOI:** 10.7759/cureus.14978

**Published:** 2021-05-12

**Authors:** Ceyda H Sablak, Rebecca M Dudley, Alexander Youngdahl, Kevin R Roth

**Affiliations:** 1 Department of Emergency and Hospital Medicine, University of South Florida Morsani College of Medicine/Lehigh Valley Health Network Campus, Allentown, USA

**Keywords:** point-of-care, ultrasound, lymphoma

## Abstract

T-cell lymphoblastic lymphoma (T-cell LBL) is an uncommon diagnosis for acute dyspnea in pediatric emergencies. This case details a 13-year-old boy presenting to the ED with dyspnea, who was diagnosed with T-cell LBL. It was a unique presentation in which there was no obvious mediastinal mass on the examination or primary imaging. As a safe and cost-effective modality for a patient that was too unstable to transfer to the radiology department for computed tomography, point-of-care ultrasound (POCUS) was useful in the patient’s rapid assessment for suspected pericardial and pleural effusion. This case highlights the advantage of early utilization of POCUS for pediatric patients with dyspnea.

## Introduction

Point-of-care ultrasound (POCUS) is a promising dynamic imaging modality that provides physicians the freedom to acquire and interpret images synchronously with clinical decision-making while remaining economical and lacking ionizing radiation [1]. With goal-directed training, physicians can accurately assess and identify suspicious changes [2]. Still, more guidelines are needed to determine when it is appropriate to use ultrasound in lieu of other modalities like CT [3]. The main indications for chest ultrasound in pediatrics include assessing an opaque hemithorax, evaluating masses and lesions, and confirming pleural effusions [4]. In febrile or afebrile dyspneic children with opaque hemithorax of chest radiograph, POCUS differentiates among pulmonary, pleural, and mediastinal lesions [4].

Cardiac POCUS focuses on identifying pericardial effusions and tamponade. POCUS is both sensitive and specific for identifying pericardial effusion and left ventricular systolic dysfunction [5]. Only one-third of patients with tamponade will present with Beck’s triad (jugular venous distention, muffled heart tones, and hypotension), and 10% will not present with any of these signs; therefore, diagnosis of tamponade can be made with POCUS before it becomes clinically apparent, potentially expediting treatment [6].

Even though biopsy is the gold standard for diagnosing lymphoma, POCUS plays a unique role in emergency settings to instantly investigate possible causes of life-threatening complications. POCUS has been shown to reduce the time to diagnosis in patients with dyspnea in the adult population [7]. Although presumptuous that this would translate to pediatric populations, unfortunately, there is limited literature regarding the use of POCUS in pediatric populations with dyspnea [8]. One case report describes POCUS revealing a mediastinal mass in a boy presenting to the ED with dyspnea for one month and an abnormal chest x-ray six months prior, who was lost to follow-up and eventually diagnosed with B-cell lymphoma [9]. Due to the severity of his symptoms and risk of cardiovascular collapse with supine positioning, CT was ultimately postponed until pericardiocentesis, illustrating how utilization of POCUS impacted clinical decision-making [9]. In our case, we present how POCUS assisted in managing an adolescent who presented with shortness of breath.

## Case presentation

**Figure 1:**
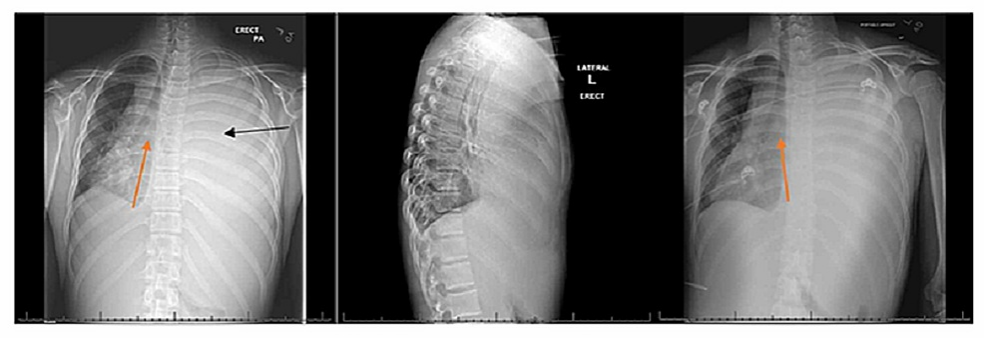
Chest x-ray performed in the ED (PA, LAT, and AP views) illustrating complete opacification of the left hemithorax (black arrow) with a significant shift of the mediastinum, and tracheal deviation, to the right (red arrows). PA = posterior-anterior, LAT = lateral, AP = anterior-posterior

A 13-year-old male presented to a rural ED with a five-day history of progressively worsening shortness of breath and a three-day history of left-sided chest wall pain and swelling. He had been sick, with, what seemed to be a cold, for the seven days prior. He reported subjective fever, nausea, and multiple episodes of vomiting. His review of systems was also positive for orthopnea, diaphoresis, and adenopathy. In the ED, he was afebrile, ill-appearing, in respiratory distress with dyspnea and accessory muscle use. He had muffled heart sounds with visible left-sided chest wall swelling. He was tachycardic and tachypneic, with stable blood pressure (BP) and oxygen saturation; his vital signs were: BP 123/89 mmHg, pulse: 126 beats/minute, temperature: 97.9°F, respiratory rate: 28 breaths/minute, and pulse oximetry of SpO2: 95%. There was complete opacification of the left hemithorax with a significant shift of the mediastinum, and tracheal deviation, to the right on chest x-ray (Figure 1).

**Figure 2:**
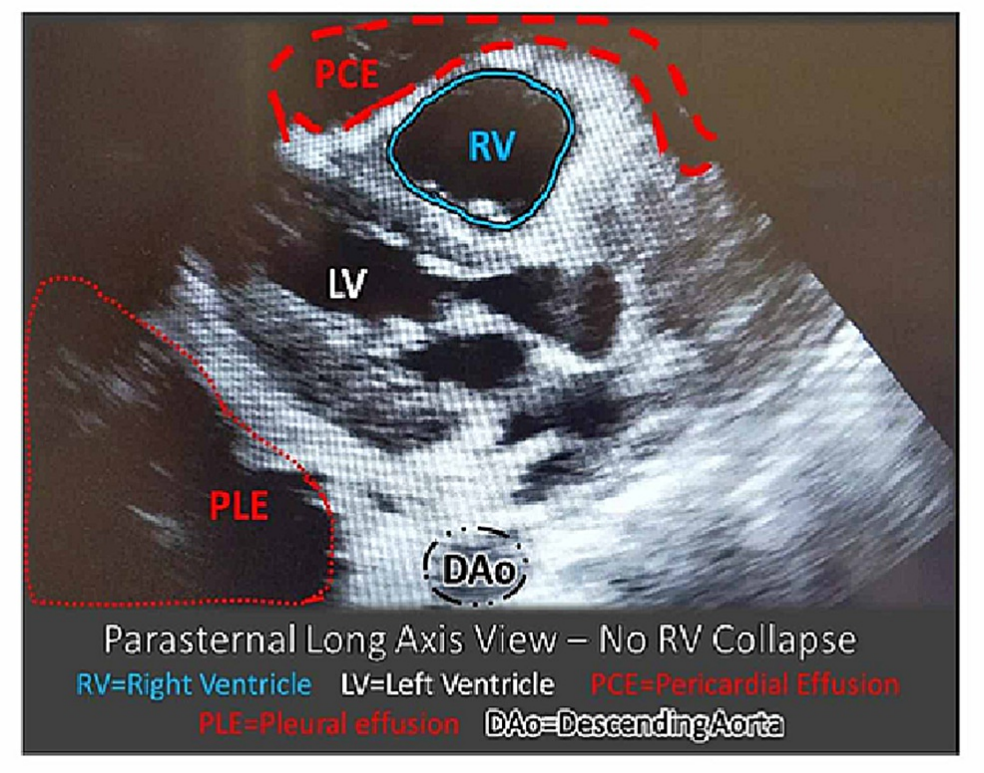
POCUS of the heart parasternal long-axis view – note the RV is not collapsed. There is a small PCE. A PLE is noted as it is posterior to the DAo. POCUS: point-of-care ultrasound; PCE: pericardial effusion; PLE: large pleural effusion; DAo: descending aorta; RV: right ventricle

**Figure 3:**
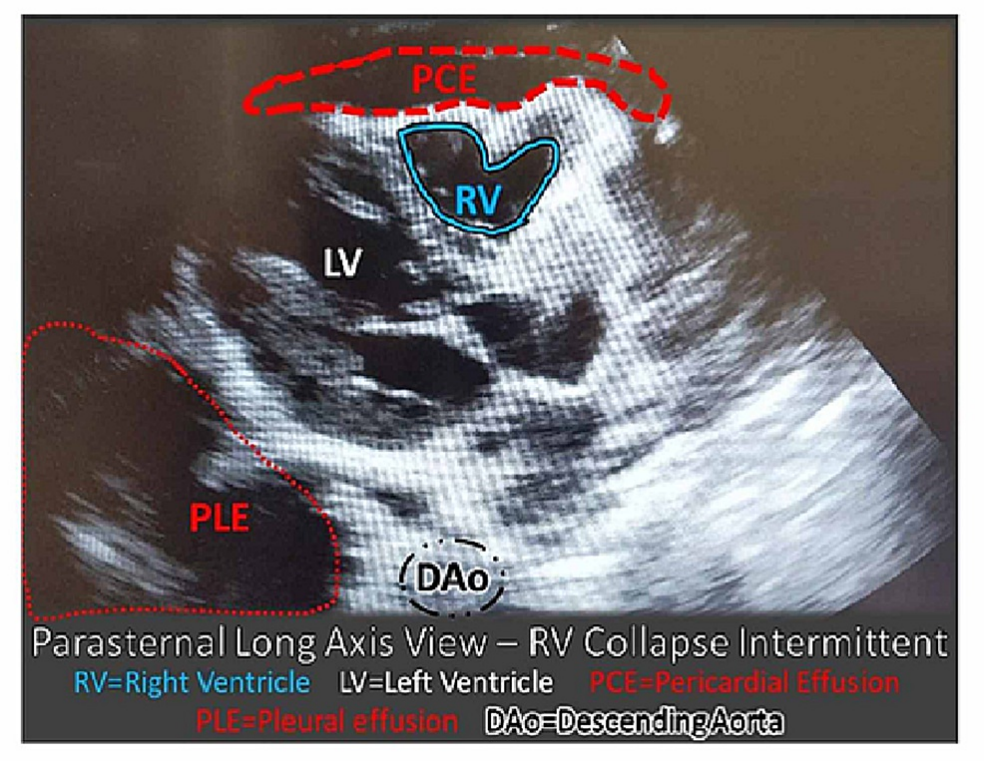
POCUS of the heart parasternal long-axis view – note the RV is collapsing in diastole. There is a small PCE. A large PLE is noted. Right ventricle collapse is not in proportion to the size of the pericardial effusion. When considered in the context of the pleural effusion, it is suggestive of a probable mass effect. POCUS: point-of-care ultrasound; PCE: pericardial effusion; PLE: large pleural effusion; DAo: descending aorta; RV: right ventricle

The right lung showed lung markings without increased vascular congestion or acute infiltrate. These findings could be indicative of atelectasis, pleural effusion, or a large pulmonary mass. Transfer to a higher-level pediatric hospital with more resources available was arranged as POCUS was done concurrently. His neck did not exhibit any compressive masses or jugular venous distention; however, POCUS of the chest revealed a large pleural effusion and small pericardial effusion, with a possible underlying mass, and hyperdynamic heart with tamponade (Figures 2, 3).

Complete blood count (CBC) revealed leukocytosis (11.7, normal 3.8-10.4 thou/cmm), thrombocytosis (604, normal 177-381 thou/cmm), mild polycythemia (5.47, normal 4.20-5.30 mill/cmm), and elevated RDW (15.6, normal 11.4-13.5%). Differential revealed elevated absolute neutrophils (9.1, normal 1.7-7.0), absolute monocytes (0.9, normal 0.1-0.6), neutrophils (77, normal 38-64), and monocytes (7, normal 3-5), as well as decreased lymphocytes (14, normal 26-43). Lactate dehydrogenase was elevated (290, normal 87-241 U/L).

In preparation for transfer and discussion with the receiving pediatric intensive care unit (PICU) team, it was decided to defer chest tube placement in the ED (unless tension physiology developed) because they were better equipped to manage complications and the possibility of biopsy was being entertained.

**Figure 4:**
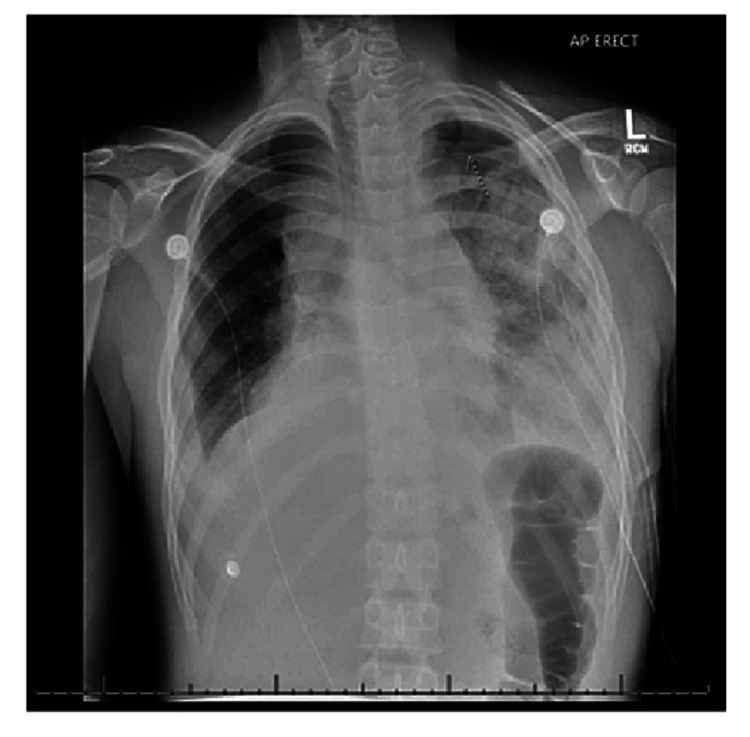
Chest x-ray (AP) view was done after the chest tube was placed and fluid drained showing enlargement of the left hilum pulmonary hilum and AP window region, raising the possibility of mediastinal mass. AP = anterior-posterior

**Figure 5:**
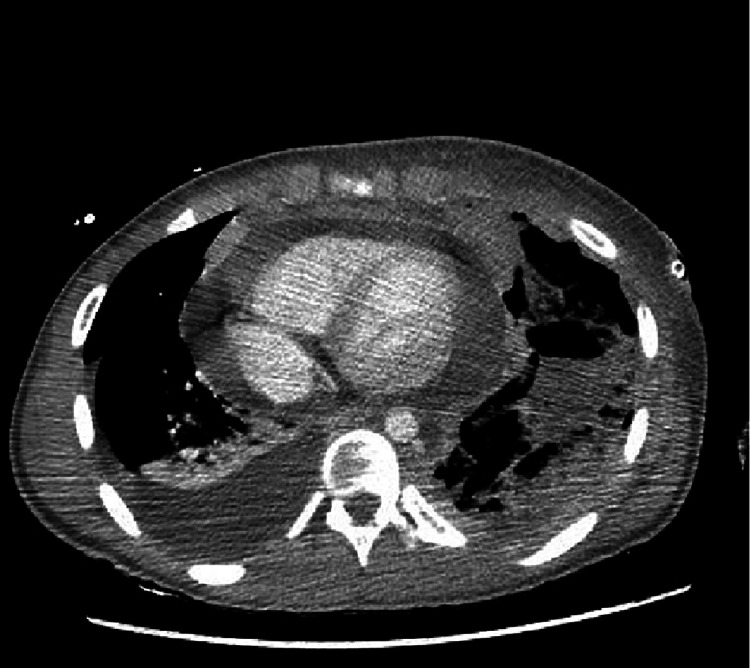
Large heterogenous mediastinal mass.

The patient was supported with 2 L O2 via nasal cannula and IV fluids and then transferred to the PICU at a tertiary center for further management. Chest tube placement by pediatric surgery at the receiving hospital provided a marked improvement in hemodynamics and respiratory status, with 2500cc of fluid initially evacuated. A repeat chest x-ray suggested a left mediastinal mass (Figure 4). This was confirmed with contrast-enhanced CT adhering to as low as reasonably achievable (ALARA) dosing principles at the tertiary hospital (Figure 5). T-cell lymphoblastic lymphoma was diagnosed via biopsy taken during chest tube placement, and the patient was started on chemotherapy. 

## Discussion

This case illustrated the importance of having a low threshold for using POCUS in patients with life-threatening complaints like dyspnea. Although T-cell LBL is an uncommon diagnosis and unlikely cause of dyspnea in an adolescent, this case demonstrates how POCUS can enhance the evaluation of dyspneic patients in the ED. Initial chest x-ray findings, with a left to right mediastinal shift and opacification of the left hemithorax, may have been due to a large left pleural effusion with mass effect on the mediastinum, with no definitive mass. A mass or neoplasm could not be excluded based on the x-ray findings. POCUS increased suspicion because the intermittent collapsing of the right ventricle was not in proportion to the small pericardial effusion. This disproportion leads to perceiving a mass effect on POCUS, which was confirmed on CT in the PICU. In this case, at a remote rural ED site, it corroborated the immediate understanding that the patient needed to be triaged to a pediatric tertiary care institution where a biopsy could be undertaken.

As a safe and cost-effective tool, guidelines regarding POCUS use in pediatric emergencies should be developed. As the only radiation-free imaging modality, it can help determine diagnoses and guide procedures such as chest tube placement, thoracentesis, pericardiocentesis, and peritoneal drainage [10,11]. Although the significance of POCUS in pediatric emergencies is well-supported in medical literature, the standards of practice and predictable routine use have yet to be defined [10-12]. T-cell LBL is a rare condition that can lead to abrupt deterioration if left untreated [13]. In this case, the advantages of POCUS were used to define the extent of pleural and pericardial effusion and increased suspicions of clinicians toward this unusual condition. One caution by the American Academy of Pediatrics is that POCUS should not rule out a suspected diagnosis, but it is a helpful modality for ruling in diagnoses, which was the circumstance in this case [12]. As further data is evaluated regarding the specificity of POCUS and evidence-based guidelines are developed, we may hope to confidently rule in diagnoses with this tool in the future and immediately focus on treatment options, minimizing radiation exposure secondary to other modalities.

The European Society of Paediatric and Neonatal Intensive Care (ESPNIC) sought to develop an international evidence-based POCUS guideline, which gave a “B” rating with “strong agreement” for using POCUS to diagnose pericardial effusion and pleural effusion, and to guide pericardiocentesis, chest tube placement, thoracocentesis, and peritoneal drainage; there was no discussion about how POCUS could find masses or assess T-cell LBL [10]. 

## Conclusions

Future research on how to encourage the use of POCUS at the bedside and demonstrate quantitative and qualitative benefits is necessary. POCUS is a safe and cost-effective tool that should be encouraged in pediatric emergencies such as shortness of breath. Assisted by POCUS, an expeditious diagnosis of pleural and pericardial effusion was made in this case.
